# Trends in the Incidence of Ovarian Cancer Among Premenopausal and Postmenopausal Women in the United States, 2001 to 2021

**DOI:** 10.3390/cancers17132119

**Published:** 2025-06-24

**Authors:** Victor Adekanmbi, Abbey B. Berenson, Batul Shakir, Christine D. Hsu, Thao N. Hoang, Itunu O. Sokale, Tolulope T. Sajobi, Fangjian Guo

**Affiliations:** 1 Center for Interdisciplinary Research in Women’s Health, School of Medicine, The University of Texas Medical Branch, Galveston, TX 77555, USA; abberens@utmb.edu (A.B.B.); cdhsu@utmb.edu (C.D.H.); thnhoang@utmb.edu (T.N.H.); faguo@utmb.edu (F.G.); 2 Department of Obstetrics and Gynecology, The University of Texas Medical Branch, Galveston, TX 77555, USA; 3 School of Medicine, The University of Texas Medical Branch, Galveston, TX 77555, USA; bashakir@utmb.edu; 4 Department of Medicine, Section of Epidemiology and Population Sciences, Baylor College of Medicine, Houston, TX 77030, USA; itunu.sokale@bcm.edu; 5 Dan L. Duncan Comprehensive Cancer Center, Baylor College of Medicine, Houston, TX 77054, USA; 6 Cumming School of Medicine, University of Calgary, Calgary, AB T2N 1N4, Canada; ttsajobi@ucalgary.ca

**Keywords:** ovarian cancer, incidence, trend, premenopausal, postmenopausal, United States

## Abstract

Background: Ovarian cancer remains the deadliest and leading cause of gynecological cancer-associated mortality in the US. The aim of this study was to characterize the trends in the incidence of ovarian cancer between premenopausal and postmenopausal women to inform future targeted interventions. Methods: This population-based cross-sectional study analyzed data from the US Cancer Statistics (USCS) database, which covered the whole of the US population between 2001 and 2021. Joinpoint regression was used to compute the average annual percentage change (APC) with 95% confidence interval (CI) and age-standardized incidence rates per 1,000,000 population. Results: The results showed that the IR of ovarian cancer declined between 2001 and 2021. Postmenopausal women had greater decreases in the IR of ovarian cancer compared to premenopausal women who showed a small decline. When stratified by race/ethnicity, non-Hispanic American Indian/Alaska Native women aged 20–49 years experienced an increase in the IR of ovarian cancer (APC = 2.4; 95% CI 0.9 to 4.1) compared to other racial/ethnic groups which showed a decline. Joinpoint trend analyses identified one inflection point in localized ovarian cancer incidence trends among all three age groups: an initial decline from 2001 to 2011 among women 20–49 years old and 65+ years old, and from 2001 to 2012 among women 50–64 years old, followed by an upward trend thereafter to 2021. Similarly, there was one inflection point in the IR of ovarian cancer for the clear cell and endometrioid types among women aged 20–49 years old. Conclusions: The IR of ovarian cancer in the US declined significantly among postmenopausal compared to premenopausal women, for whom the IR of ovarian cancer decreased only slightly. Although encouraging, these findings show a need for continued efforts to improve early detection and prevention strategies to mitigate the burden of this deadly disease.

## Simple Summary

In the United States (US), ovarian cancer is the most fatal of all the gynecological cancers. We conducted a nationwide analysis of data spanning the period from 2001 to 2021 to examine trends in ovarian cancer incidence among premenopausal and postmenopausal women, with the aim of informing future targeted interventions. Our findings showed a significant decline in the incidence rate (IR) of ovarian cancer among postmenopausal women, while the decline among premenopausal women was only slight. These results highlight the need for continued concerted efforts to improve early detection and prevention strategies to reduce the burden of ovarian cancer in the US.

## 1. Introduction

Ovarian cancer is the leading cause of female reproductive system cancer-associated mortality in the US, with an estimated 19,860 new cases and 12,740 deaths projected in 2024 [1]. The high mortality rate is largely due to the difficulty in early detection, which is linked to the tumor’s asymptomatic growth, delayed onset of symptoms, and limited accuracy due to the low sensitivity and specificity of the available screening methods [2]. As a result, over 70% of patients are diagnosed at stage III or IV (advanced stage), leading to poor prognosis even with immediate and aggressive treatment [3].

The overall incidence of ovarian cancer in the US declined from 1975 to 2018, with rates decreasing across various racial groups [3]. However, recent data from 2020 reveals a concerning trend; while global ovarian cancer incidence has generally decreased, there is a notable increase in incidence among younger women [4]. For instance, in the US, ovarian cancer rates are increasing in African American women aged 40 or younger [4]. Although ovarian cancer primarily affects postmenopausal women, the recent upward trend among younger women may be due to increasing rates of obesity, diabetes, metabolic syndrome, unopposed estrogen exposure, and nulliparity [4,5]. Furthermore, previous studies indicated a higher prevalence of genetic testing for BRCA1 and BRCA2 mutations in women aged 40 or younger, which are associated with an elevated risk of ovarian cancer [6,7,8]. Conversely, postmenopausal women experience different risk profiles, with family history, high-fat diets, delayed menopause, and the use of hormone replacement therapy emerging as key risk factors [9,10]. Understanding how these risk factors evolve with age and interact with changing lifestyle and healthcare is crucial for interpreting the temporal shifts in ovarian cancer incidence.

Previous studies have primarily focused their attention on global patterns and trends, with their analysis addressing overall ovarian cancer rates, histologic subtypes, and demographic variations [11]. While studies conducted in the US have explored ovarian cancer trends across histotypes and race/ethnic groups, to our knowledge, no studies to date have examined the distinction between premenopausal and postmenopausal incidence rates (IRs) [12,13]. Addressing this gap in the literature will provide a better understanding of how trends in ovarian cancer incidence impact different age groups and menopausal status over the years. This will help in the development of risk prediction algorithms that can improve the performance of diagnostics for ovarian cancer. The overarching aim of this study was to examine temporal trends in ovarian cancer incidence among premenopausal and postmenopausal women in the US over a 20-year period from 2001 to 2021.

## 2. Materials and Methods

### 2.1. Study Population and Data

This study used data from the United States Cancer Statistics (USCS) database [14]. This database compiles information from two primary sources: the National Program for Cancer Registries (NPCR), managed by the Centers for Disease Control and Prevention (CDC), a US federal agency; and the Surveillance, Epidemiology, and End Results (SEER) Program. These cancer registries gather demographic and tumor-specific data from hospitals, healthcare providers, and laboratories across all 50 states and the District of Columbia, providing comprehensive cancer incidence and population data covering nearly the entire US adult population.

The study population included all females 20 years and older in the US. We included cases of microscopically confirmed, malignant ovarian cancer, as defined by the International Classification of Diseases for Oncology, Third Edition (ICD-O-3). Eligible cases were required to have a primary tumor site code of C56.9 (ovary). Cases that were identified only by autopsy or death certificate were all excluded. Histologic subtypes were classified based on ICD-O-3 codes and included clear cell (8290, 8310, 8313, 8443, and 8444), endometrioid (8380–8383, 8482, and 8570), mucinous (8470–8472, 8480, 8481, and 9015), and serous (8050, 8120–8122, 8120, 8130, 8260, 8441, 8442, 8450, 8460–8463, and 9014) [15]. Cases were further categorized by their stage at diagnosis as localized, regional, or distant.

Because cancer registries do not typically collect data on menopausal status, we used age at diagnosis as a proxy and categorized it into the following age groups: 20–49 years old (premenopausal), 50–64 years old, and 65 years and older (postmenopausal). We chose to categorize the postmenopausal age group into two categories of 50–64 years and 65+ years for the comprehensive interpretation of the results. Geographic regions of residence were categorized as South, Northeast, Midwest, and West. Race and ethnicity were classified into Hispanic, Non-Hispanic Black (NHB), Non-Hispanic White (NHW), Non-Hispanic Asian or Pacific Islander (NHAPI), and Non-Hispanic American Indian/Alaska Native (NHAIAN).

### 2.2. Statistical Analysis

To examine variations in ovarian cancer IRs in the US over the past 20 years, we fitted Joinpoint regression models [16] using annual incidence data spanning the period from 2001 to 2021 [17]. We estimated the IRs of ovarian cancer as cases per 1,000,000 persons. We further age-adjusted the IR to the 2000 US standard population. Subgroup analyses were performed for age groups based on menopausal status and race/ethnicity. In accordance with data confidentiality requirements, statistics were not reported when the case count in a cell was less than 16; these values were treated as missing values in our analysis. Joinpoint regression analysis was used to detect significant changes in ovarian cancer incidence trends. This method fits a series of log-linear models to the data to identify specific years (joinpoints) where the annual percentage change (APC) shifts significantly and determines the minimum number of joinpoints that best fit the annual data. Statistical significance for all two-sided tests was set at *p* < 0.05. The Tiwari method [18] was used to compute confidence interval (CI). APCs used the formula (exp[β] − 1) × 100, where the regression coefficient (β) was derived from a least-squares regression of the natural logarithm of the rates against the calendar year. Methods developed by Kleinbaum and colleagues [19] were used to test the statistical significance of APCs as well as variations between APCs. All analysis were conducted using SEER*Stat (version 8.4.3) and the Joinpoint regression analysis program for Windows (version 5.2.0) [17].

## 3. Results

From 2001 to 2021, there were 444,865 cases of invasive ovarian cancer among adult women ≥ 20 years old. Of these, 80,212 (18.0%) cases occurred in women aged 20–49 years, 150,371 (33.8%) cases in women aged 50–64 years, and 214,282 (48.2%) cases in women aged 65 years and older. In 2021, NHB women had the lowest incidence rate among those aged 20–49 years old (50.2 per 1,000,000) and 50–64 years old (185.2 per 1,000,000). Among women aged 65 years and older, NHAPI women had the lowest incidence rate (206.1 per 1,000,000; Table 1). On the other hand, NHAPI had the highest IR in the 20–49 years (66.7 per 1,000,000) and 50–64 years (232.9 per 1,000,000) while NHAIAN had the highest IR in the 65 years and older group (389.9 per 1,000,000).

**Figure 1:**
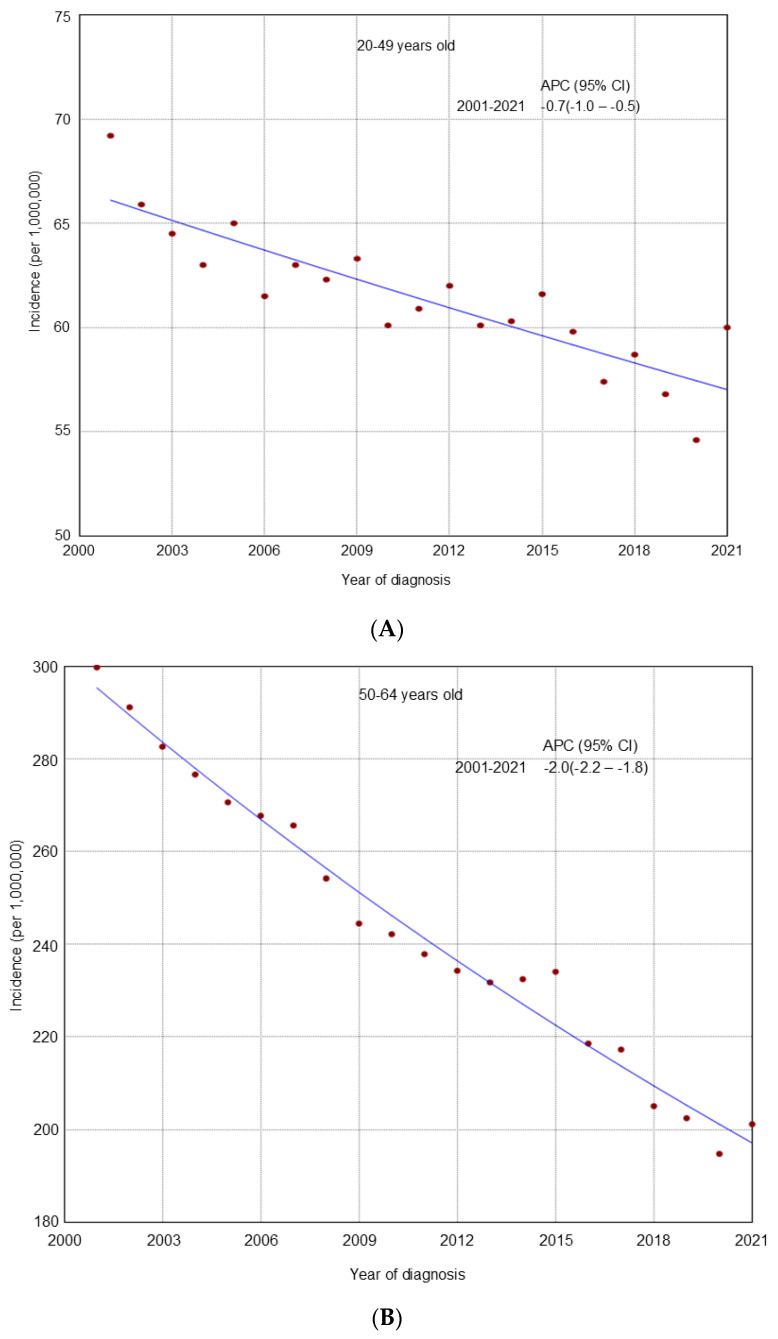

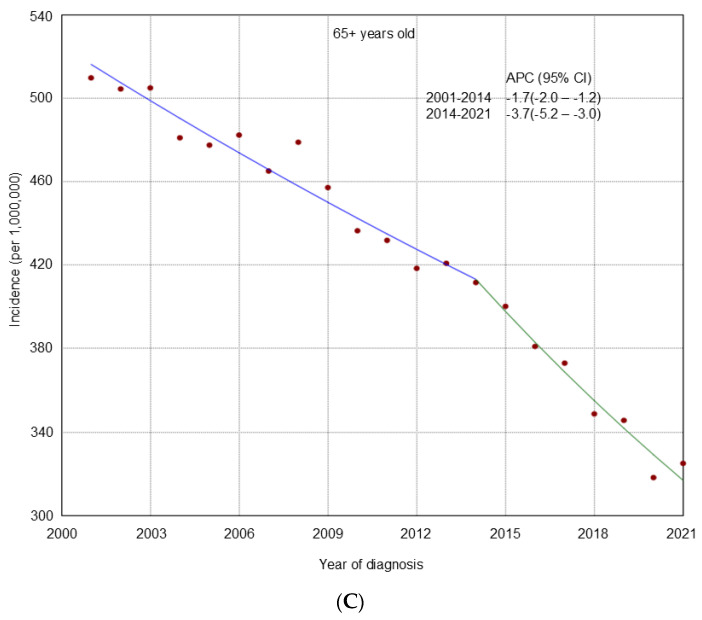
**Age-specific trends in** ovarian cancer incidence among U.S. women aged 20 years and older, 2001–2021. (**A**). 20–49 years age group; (**B**): 50–64 years age group; (**C**): 65 years and older age group.

**Figure 2:**
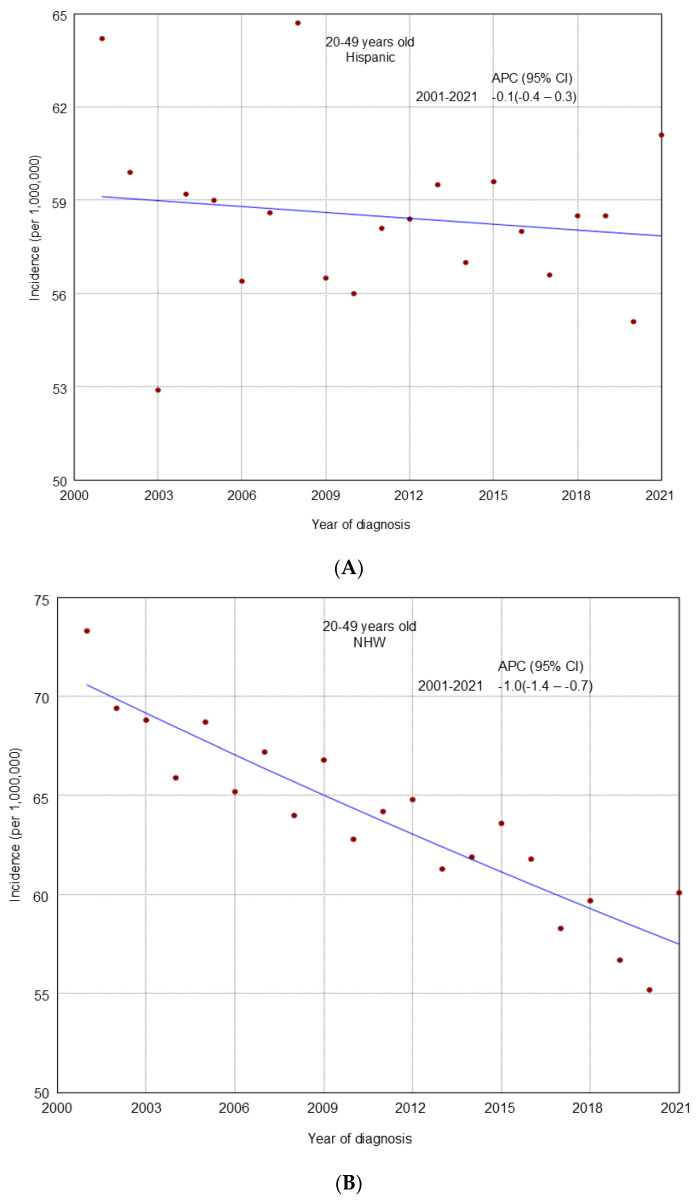

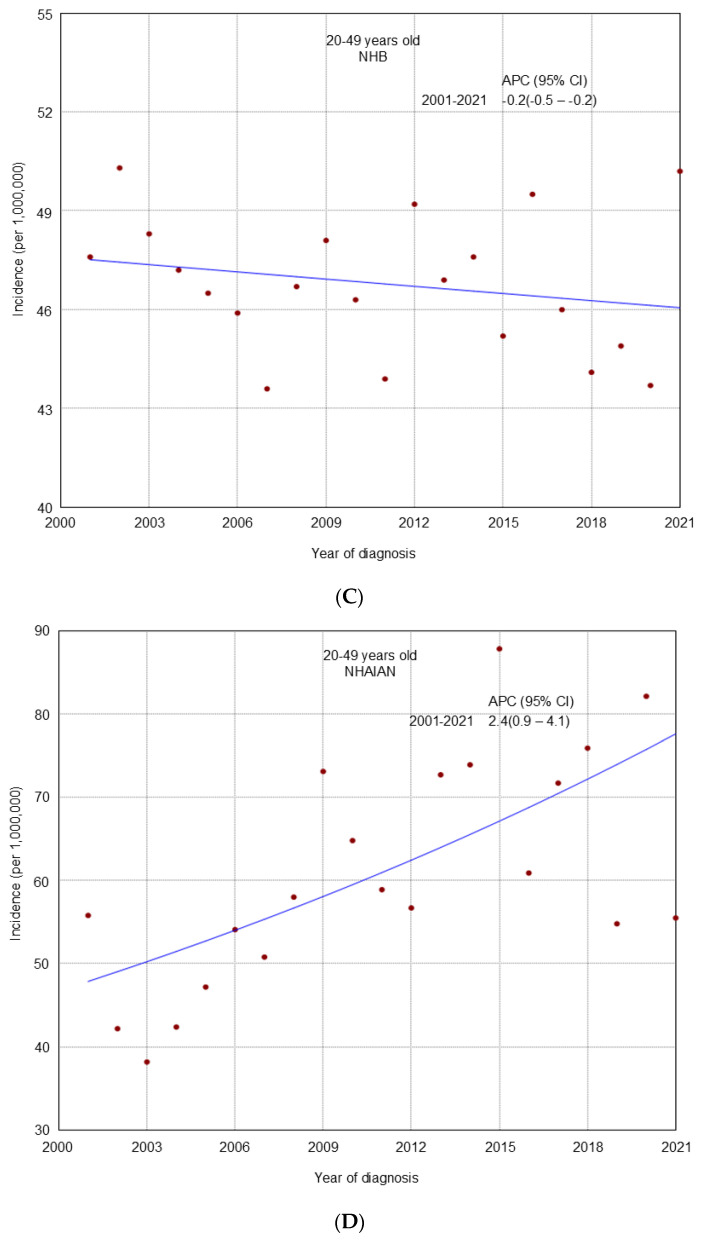

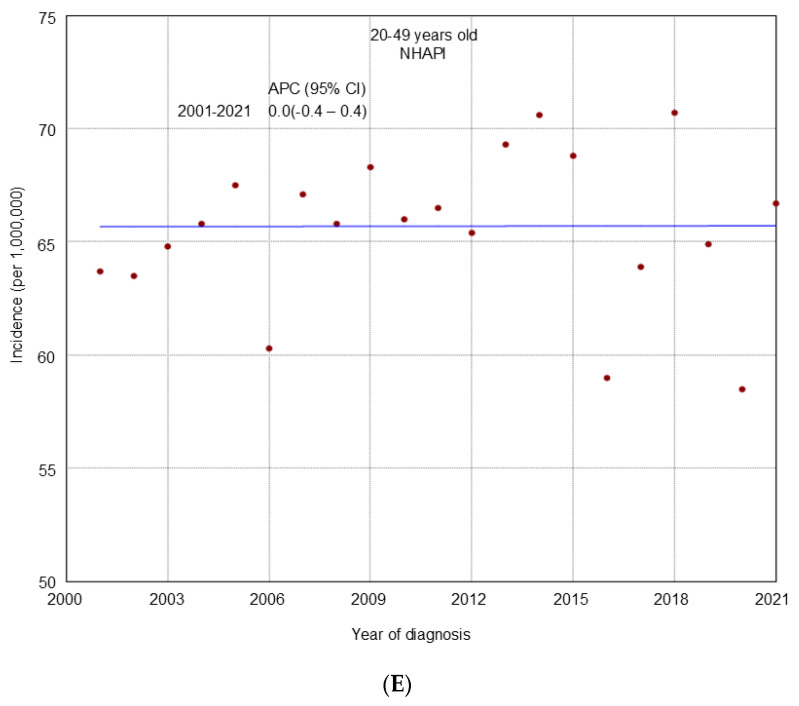
Trends in ovarian cancer incidence from 2001 to 2021 among U.S. women aged 20-49 years stratified by race and ethnicity. (**A**): Hispanic; (**B**): Non-Hispanic White (NHW); (**C**): Non-Hispanic Black (NHB); (**D**): Non-Hispanic American Indian/Alaska Native (NHAIAN); (**E**): Non-Hispanic Asian or Pacific Islander (NHAPI).

**Figure 3:**
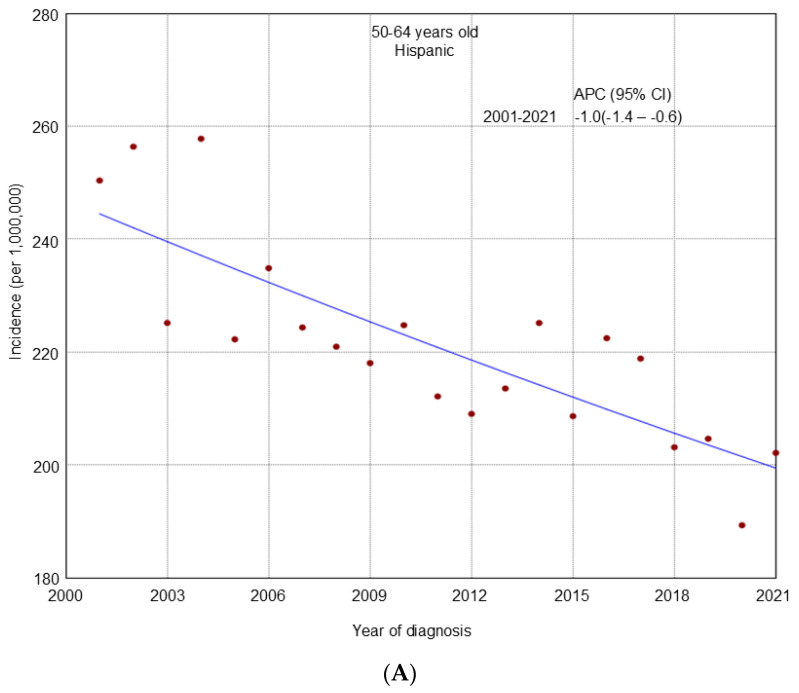

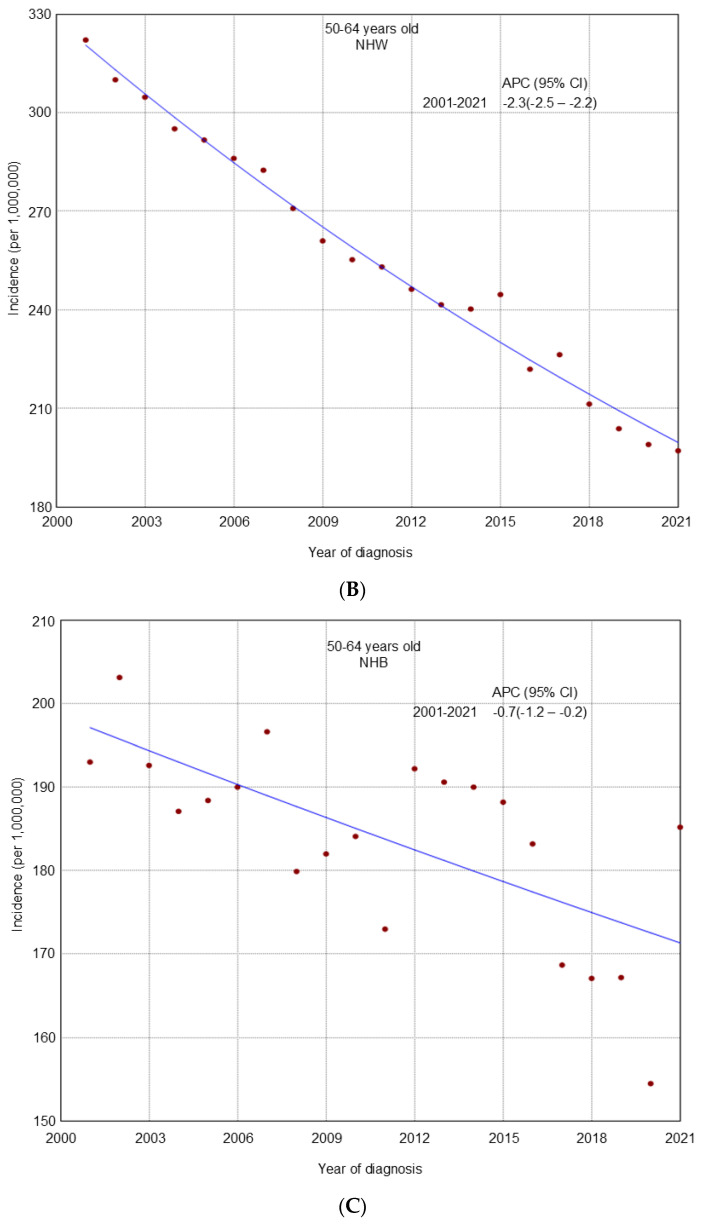

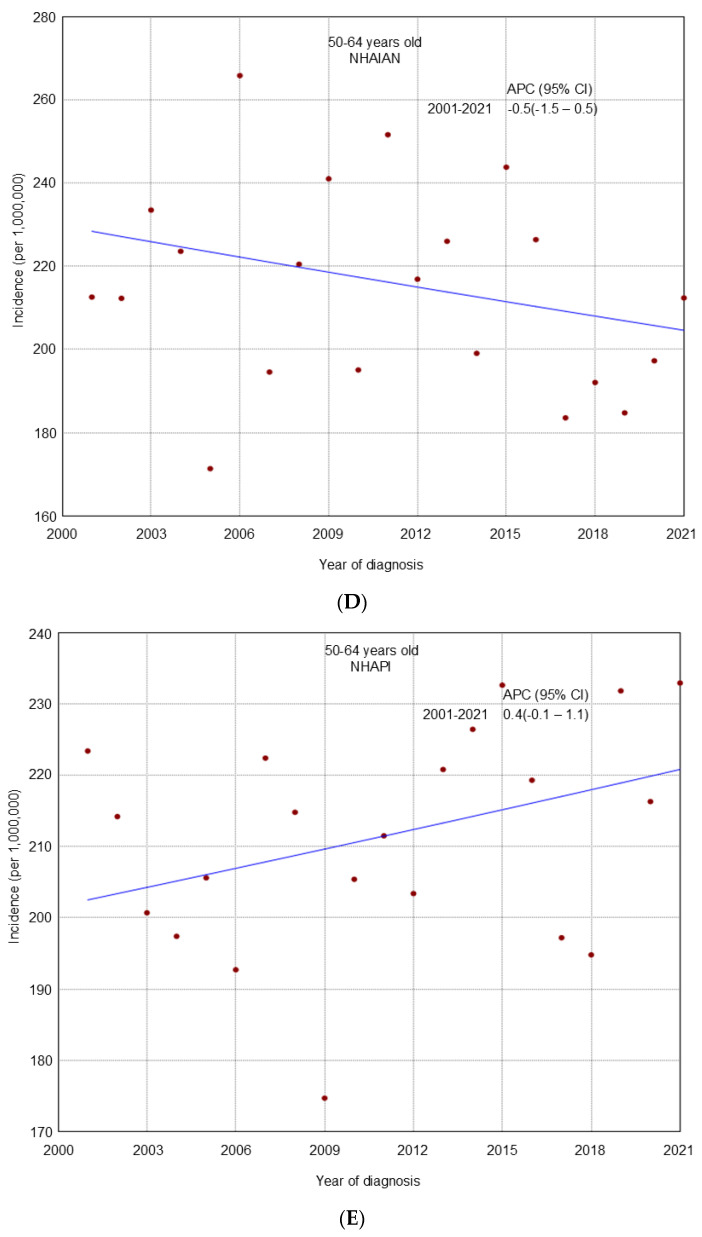
Trends in ovarian cancer incidence from 2001 to 2021 among U.S. women aged 50-64 years stratified by race and ethnicity. (**A**): Hispanic; (**B**): Non-Hispanic White (NHW); (**C**): Non-Hispanic Black (NHB); (**D**): Non-Hispanic American Indian/Alaska Native (NHAIAN); (**E**): Non-Hispanic Asian or Pacific Islander (NHAPI).

**Figure 4:**
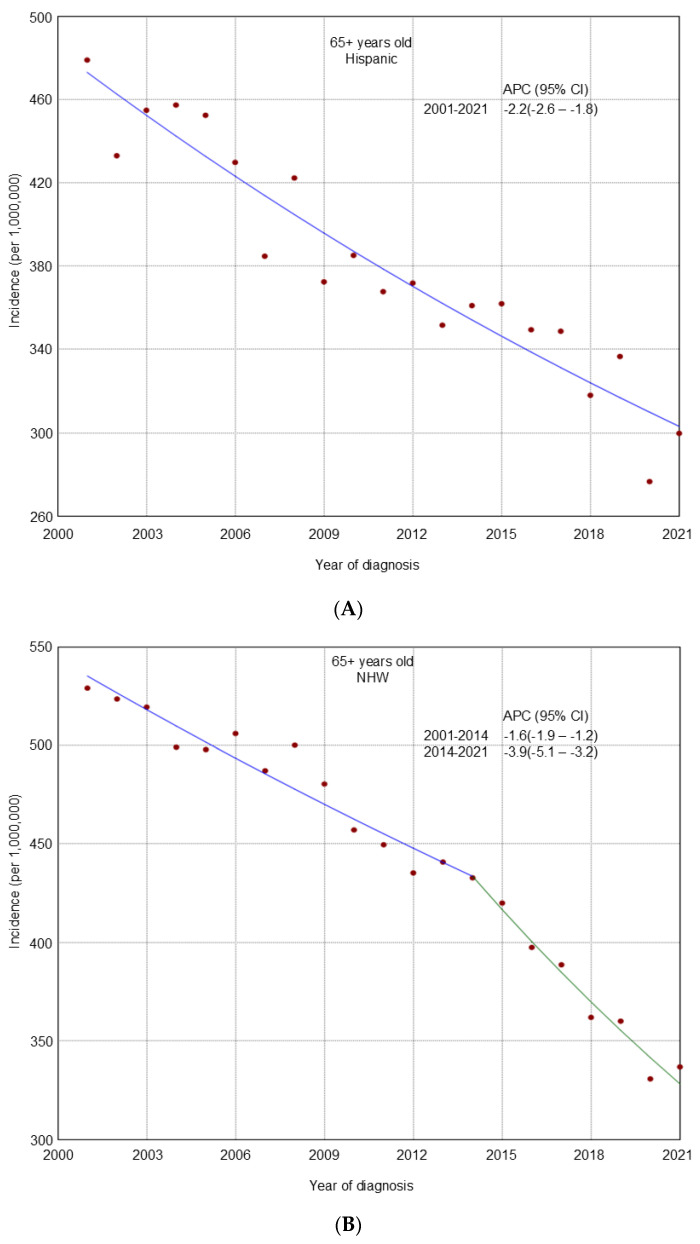

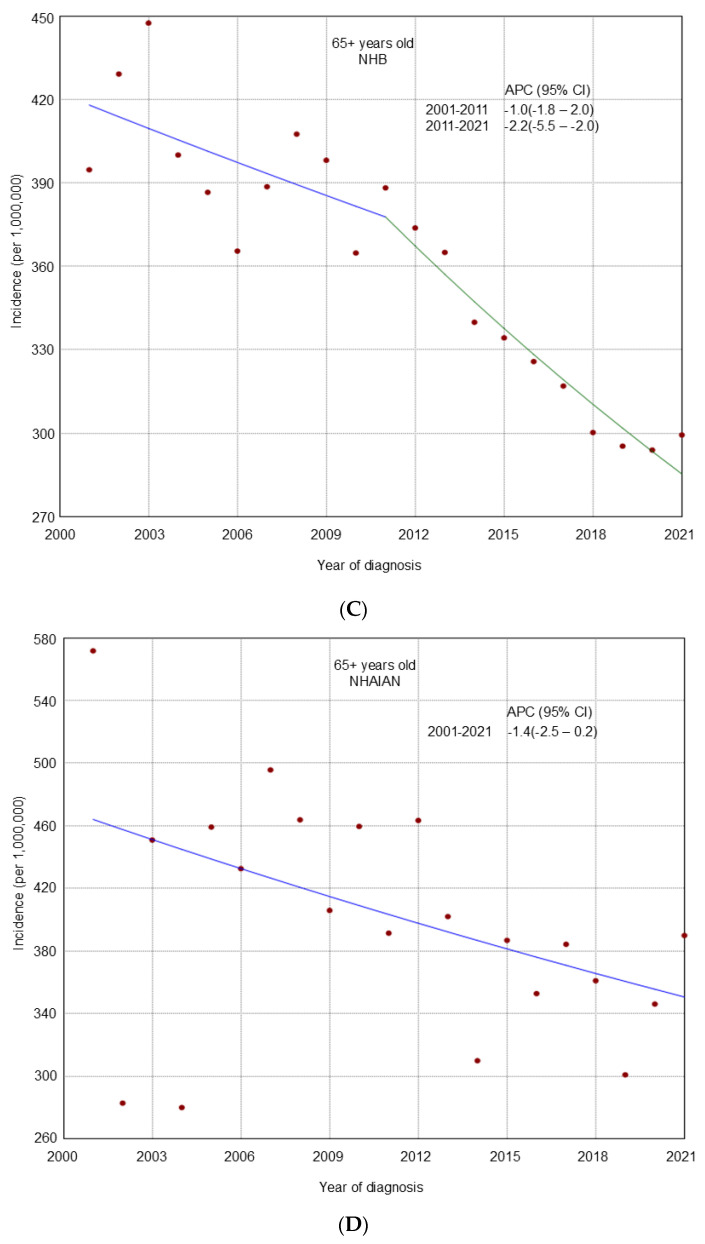

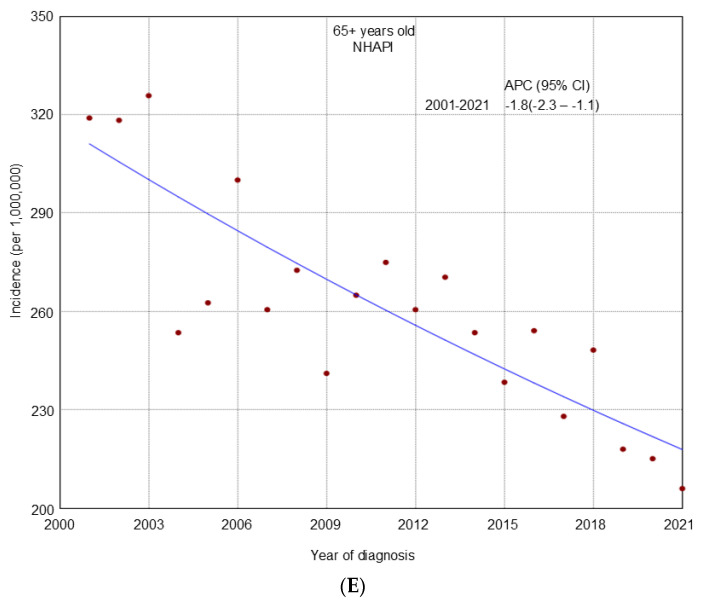
Trends in ovarian cancer incidence from 2001 to 2021 among U.S. women aged 65 years and older stratified by race and ethnicity. (**A**): Hispanic; (**B**): Non-Hispanic White (NHW); (**C**): Non-Hispanic Black (NHB); (**D**): Non-Hispanic American Indian/Alaska Native (NHAIAN); (**E**): Non-Hispanic Asian or Pacific Islander (NHAPI).

From 2001 to 2021, an overall declining trend in incidence was observed across all age groups (Figure 1). A similar decline was noted in most racial and ethnic groups by age (Figure 2, Figure 3 and Figure 4), cancer stage by age (Supplemental Figures S1–S3), and histologic subtype by age (Supplemental Figures S4–S6). Ovarian cancer incidence was relatively stable among NHAPI women in the 20–49 years old group (APC 0.0; 95% CI −0.4 to 0.4). However, increasing trends were observed among NHAIAN women aged 20–49 years (APC 2.4, 95% CI 0.9 to 4.1) and among those aged 50–64 years old (APC 0.4; 95% CI −0.1 to 1.1). Joinpoint analyses identified one joinpoint in localized ovarian cancer incidence trends among all three age groups. Among women aged 20–49 and 65 years and older, rates declined from 2001 to 2011, while among those aged 50–64, the decline extended to 2012. This was followed by an upward trend through 2021. Between 2011 and 2021, premenopausal women (20–49 years) consistently showed a higher incidence of localized ovarian cancer compared to postmenopausal groups. Additionally, one joinpoint was identified in the incidence trends of clear cell and endometrioid ovarian cancer subtypes among women aged 20–49 years, showing an initial decline followed by an increase.

## 4. Discussion

We examined the temporal trend in the incidence of ovarian cancer by age groups/menopausal status using the USCS 2001 to 2021 database. Our study revealed that the overall incidence of ovarian cancer declined among adult women from 2001 to 2021. The decline in the overall incidence of ovarian cancer seen in this study could be linked to the increased adoption of risk-reducing surgeries and combined oral contraceptive use as preventive strategies in high-risk individuals and germline mutation carriers [20,21,22,23]. Another reasonable explanation for the observed decline in the overall incidence of ovarian cancer in the US over the years could be a significant reduction in the use of postmenopausal hormone replacement therapy (HRT), particularly estrogen-alone HRT [24], that has been linked with the occurrence of ovarian cancer [25,26]. Available evidence [27,28] indicates that the incidence of HRT use declined sharply in the US in 2002 after the Women Health Initiative (WHI) large-scale randomized clinical trial associated postmenopausal HRT use with an enhanced risk of developing stroke, cardiovascular disease, dementia, and breast cancer [29]. It is, however, not clear how much of the decline in ovarian cancer incidence rate can be attributed to HRT use.

The rate of decline in the IR of ovarian cancer among premenopausal women is small compared to that of postmenopausal women. This finding reveals a remarkable shift in the landscape of ovarian cancer, highlighting its significance and burden among young and reproductive-age women. The trend pattern seen among premenopausal women might be due to the increasing prevalence of obesity, metabolic syndrome, and nulliparity among young and reproductive-age women [6,30,31,32].

Furthermore, our study found that 18% of the study population between 2001 and 2021 were diagnosed with early-onset ovarian cancer in the US, which is greater than previously reported. Prior studies [33,34] have reported a lower incidence of ovarian cancer among premenopausal women. Future studies are needed to examine the underlying factors predicting the smaller decline in the IR of ovarian cancer among premenopausal compared to postmenopausal women over the past 20 years.

We found an increasing trend in the incidence of early-onset ovarian cancer among NHAIAN women from 2001 to 2021, despite observing a decline in IRs among women from other race and ethnic groups. This finding is consistent with evidence from a previous study that analyzed the SEER 13 registry database of the NCI which covers 14.0% of the US population from 2000 to 2013 [13]. A reasonable explanation for this finding could be of the low rate of combined oral contraceptive use, decreasing parity, and the high rate of obesity that are becoming more common among NHAIAN women [13,35,36].

Moreover, our study found a rising trend in the incidence of clear cell ovarian carcinoma among premenopausal women compared to postmenopausal women. Changes in risk factor prevalence or rising genetic screening practices among younger women may likely explain this observation. The prevalence of endometriosis, a known risk factor of clear cell carcinoma of the ovary, has been found to be rising among younger women compared to older women [37].

When we restricted our data to 2021 and analysis was performed by race/ethnicity stratified by menopausal status, the highest IR of early-onset ovarian cancer was seen among NHAPI women. Similarly, NHAPI women had the highest IR of ovarian cancer among postmenopausal women aged 50–64 years, but NHAIAN women had the highest IR of ovarian cancer among women aged 65+ years. Future studies are needed to fully understand the reasons for these racial/ethnic disparities in the incidence of ovarian cancer in the US.

In 2021, we observed the highest IR of ovarian cancer in localized cases compared to regional and distant/metastasized cases when the analysis was performed by stage of disease among premenopausal women. This finding may be due to an increased adoption of risk-reducing surgery among this age group, leading to an increased number of diagnoses of early-stage ovarian cancer [20]. Although there are no standard screening methods for ovarian cancer, recent advances in imaging technology and tumor biomarker discovery, such as cell-free Deoxyribonucleic acid (DNA) methylation analysis and cancer antigen (CA)-125, have helped in identifying early-stage ovarian cancer more accurately [38].

### Strengths and Limitations

One important strength of this research work is that we used large-sample-size data from the USCS database, which covers the entire population of the US. The findings can therefore be generalized to the entire population of the US and other similar settings. Another strength of this research work is that the data used was the latest available from a comprehensive and reliable cancer registry that accurately records and categorizes all newly diagnosed cancer cases in the US.

This study has some important limitations that should be considered when interpreting the results. First, the USCS database does not have data on clinical risk factors for ovarian cancer, which meant that we were not able to adjust for HRT, family history, obesity, tobacco smoking, etc. Second, there is the potential for misclassification bias and underreporting, especially among minority ethnic groups and underserved populations. Lastly, using age as a proxy for menopausal status could lead to misclassification bias since age at menopause can often range from 45 to 55. There is a need for the cautious interpretation of the findings from this study.

## 5. Conclusions

The IR of ovarian cancer in the US declined overall, especially among postmenopausal women. Premenopausal women also showed a decline, but much less than older women. These findings underscore the need for continued efforts to improve early detection and prevention strategies to mitigate the burden of ovarian cancer.

## Figures and Tables

**Table 1:** Age adjusted ovarian cancer incidence among premenopausal and postmenopausal women in the US in 2021 by age group, race/ethnicity, histologic types, and region of residence.

Characteristics	Premenopausal (20–49)		Postmenopausal (50–64)		Postmenopausal (65+)	
	Count	Rate (95% CI)	Count	Rate (95% CI)	Count	Rate (95% CI)
**Race/ethnicity**						
Hispanic	749	61.1 (56.8–65.7)	907	202.2 (189.3–215.9)	820	299.8 (279.3–321.3)
Non-Hispanic White	2014	60.1 (57.5–62.7)	4225	197.2 (191.1–203.5)	7523	337.0 (329.4–344.8)
Non-Hispanic Black	425	50.2 (45.5–55.2)	786	185.2 (172.3–198.9)	919	299.4 (279.9–319.8)
Non-Hispanic American Indian/Alaska Native	28	55.5 (36.7–80.3)	55	212.4 (158.9–278.2)	74	389.9 (304.5–491.9)
Non-Hispanic Asian or Pacific Islander	331	66.7 (59.7–74.3)	466	232.9 (212.1–255.2)	329	206.1 (184.1–230.0)
**Histologic type**						
Serous	1055	18 (16.9–19.1)	2996	90.5 (87.2–93.8)	5054	169.7 (165.0–174.5)
Endometrioid	590	10.1 (9.3–11.0)	784	25.6 (23.8–27.5)	497	16.5 (15.0–18.0)
Clear cell	285	4.9 (4.3–5.5)	556	17.9 (16.4–19.4)	363	11.8 (10.6–13.0)
Mucinous	368	6.0 (5.4–6.6)	360	11.6 (10.4–12.8)	315	10.3 (9.2–11.5)
**Stage**						
Localized	1618	26.8 (25.5–28.2)	1819	58.3 (55.6–61.1)	1368	45.2 (42.8–47.7)
Regional	772	13.0 (12.1–13.9)	1360	42.5 (40.2–44.9)	1599	53.3 (50.7–56.0)
Distant	1029	17.5 (16.4–18.6)	3053	92.2 (88.9–95.6)	5903	198.4 (193.3–203.6)
**Region of Residence**						
Northeast	671	64.7 (59.9–69.9)	1236	202.9 (191.5–214.8)	1918	338.1 (323.0–353.7)
Midwest	617	56.6 (52.2–61.2)	1204	191.1 (180.2–202.6)	1925	329.1 (314.4–344.3)
South	1424	60.1 (57–63.3)	2447	193.4 (185.6–201.3)	3709	319.1 (308.9–329.7)
West	871	59.3 (55.4–63.4)	1615	221.6 (210.7–232.9)	2167	320.9 (307.4–334.8)
**Rural/Metro**						
Metro	3152	60.1 (58.1–62.3)	5684	204.9 (199.5–210.4)	8265	329.4 (322.2–336.6)
Rural	431	59.4 (53.9–65.3)	818	179.9 (167.3–193.2)	1454	302.5 (287.0–318.6)

Incidence rates were reported as number of cases per 1,000,000 persons and were age-adjusted to the 2000 US standard population. CI: Confidence Interval.

## Data Availability

The data utilized in this research work were obtained from the publicly accessible USCS public use database, available through the CDC’s website, which outlines specific procedures for gaining access to the data. Details of the analytical methods used in this study are available upon request.

## Supplementary Materials

The following supporting information can be downloaded at: https://www.mdpi.com/article/10.3390/cancers17132119/s1, Figure S1: Adjusted incidence rates of ovarian cancer among adult women 20–49 years old in the US from 2001 to 2021 by stage at diagnosis.; A. Localized. B. Regional. C. Distant. Figure S2: Adjusted incidence rates of ovarian cancer among adult women 50–64 years old in the US from 2001 to 2021 by stage at diagnosis. A. Localized. B. Regional. C. Distant. Figure S3: Adjusted incidence rates of ovarian cancer among adult women 65 years and older in the US from 2001 to 2021 by stage at diagnosis. A. Localized. B. Regional. C. Distant. Figure S4: Adjusted incidence rates of ovarian cancer among adult women 20–49 years old in the US from 2001 to 2021 by histologic type. A. Clear cell. B. Endometrioid. C. Mucinous. D. Serous. Figure S5: Adjusted incidence rates of ovarian cancer among adult women 50–64 years old in the US from 2001 to 2021 by histologic type. A. Clear cell. B. Endometrioid. C. Mucinous. D. Serous. Figure S6: Adjusted incidence rates of ovarian cancer among adult women 65 years and older in the US from 2001 to 2021 by histologic type. A. Clear cell. B. Endometrioid. C. Mucinous. D. Serous.
