# Supplementary material for: Trends in the Incidence of Ovarian Cancer Among Premenopausal and Postmenopausal Women in the United States, 2001 to 2021
Source: Cancers (Basel). 2025 Jun 24;17(13):2119. doi: 10.3390/cancers17132119 (PMC12249250; doi:10.3390/cancers17132119)

Supplemental Figure 1. A.

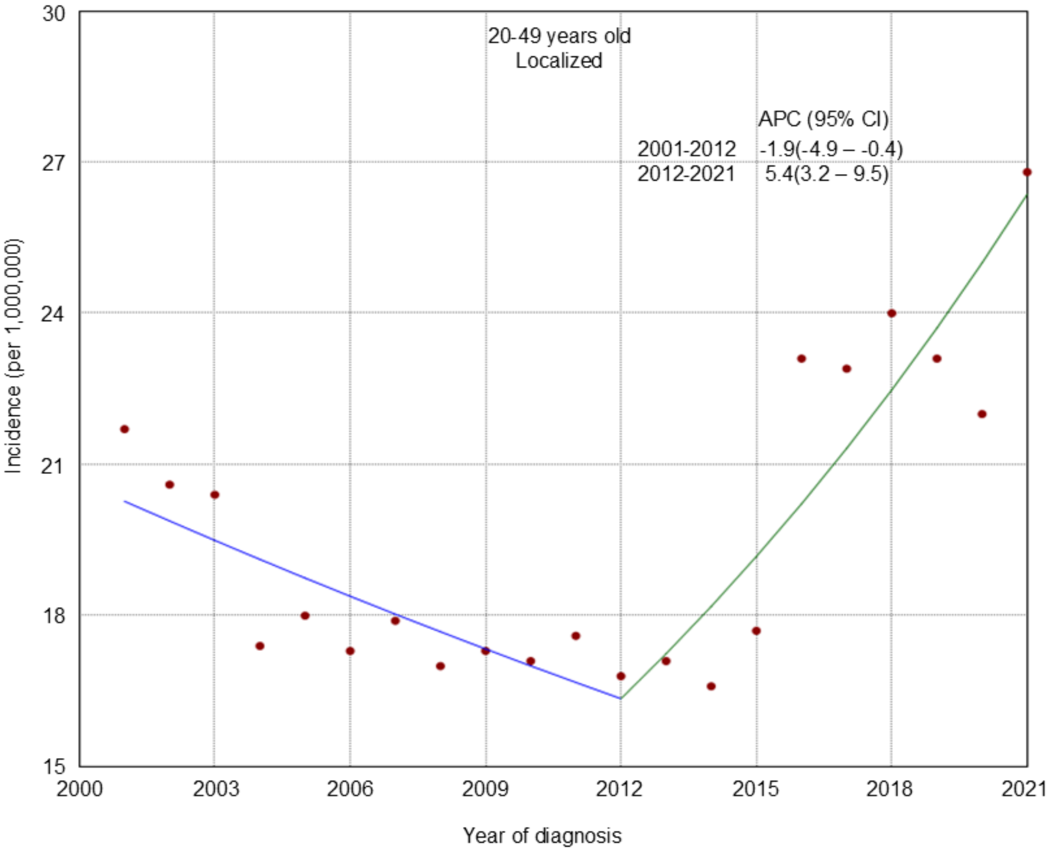

Supplemental Figure 1. B.

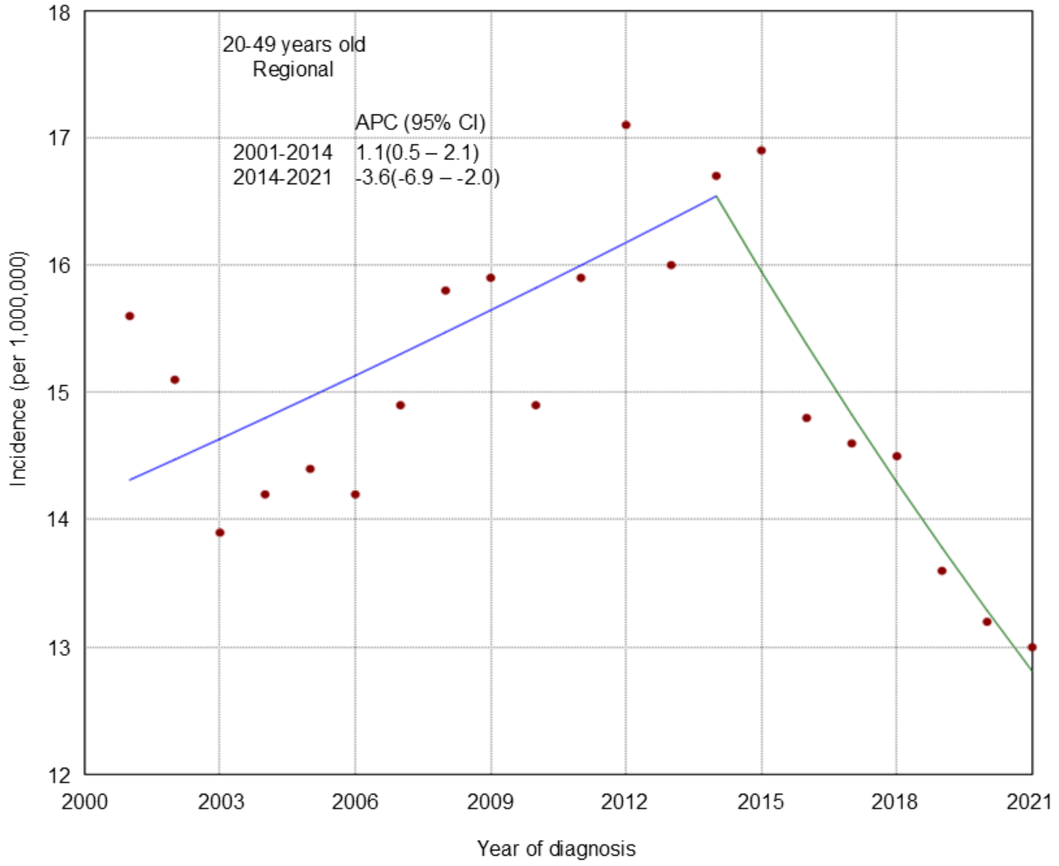

Supplemental Figure 1. C.

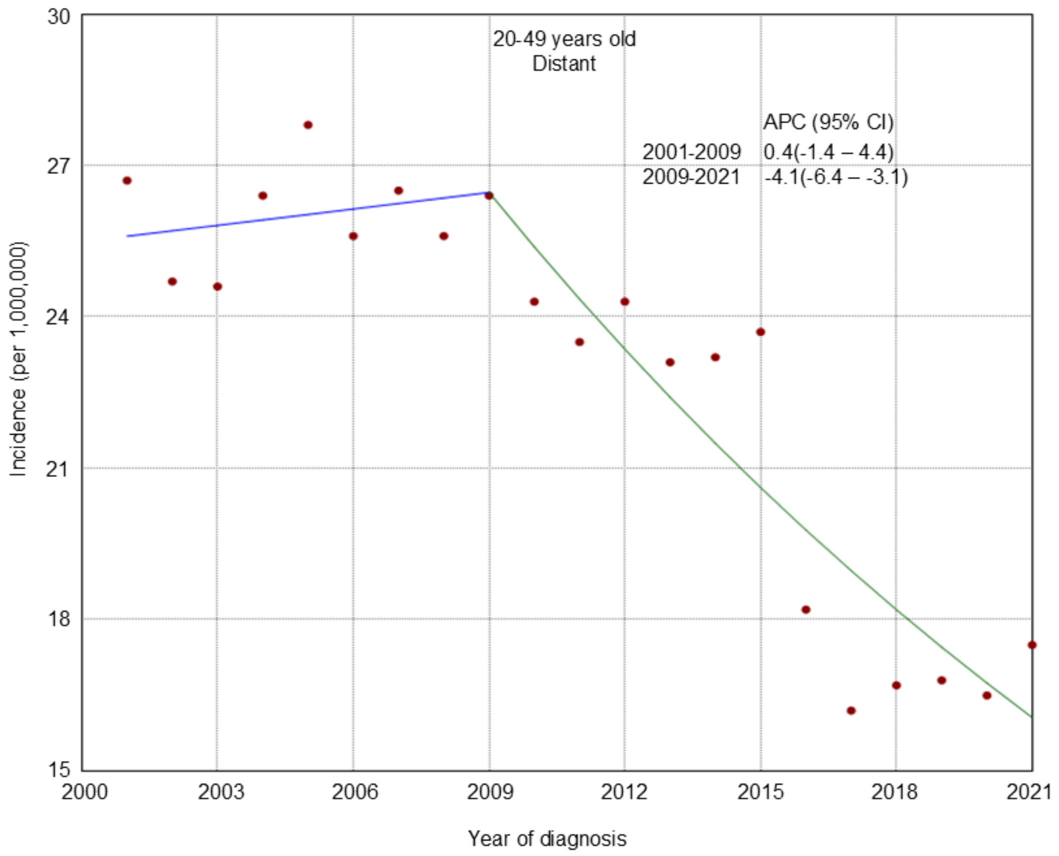

Supplemental Figure 2. A.

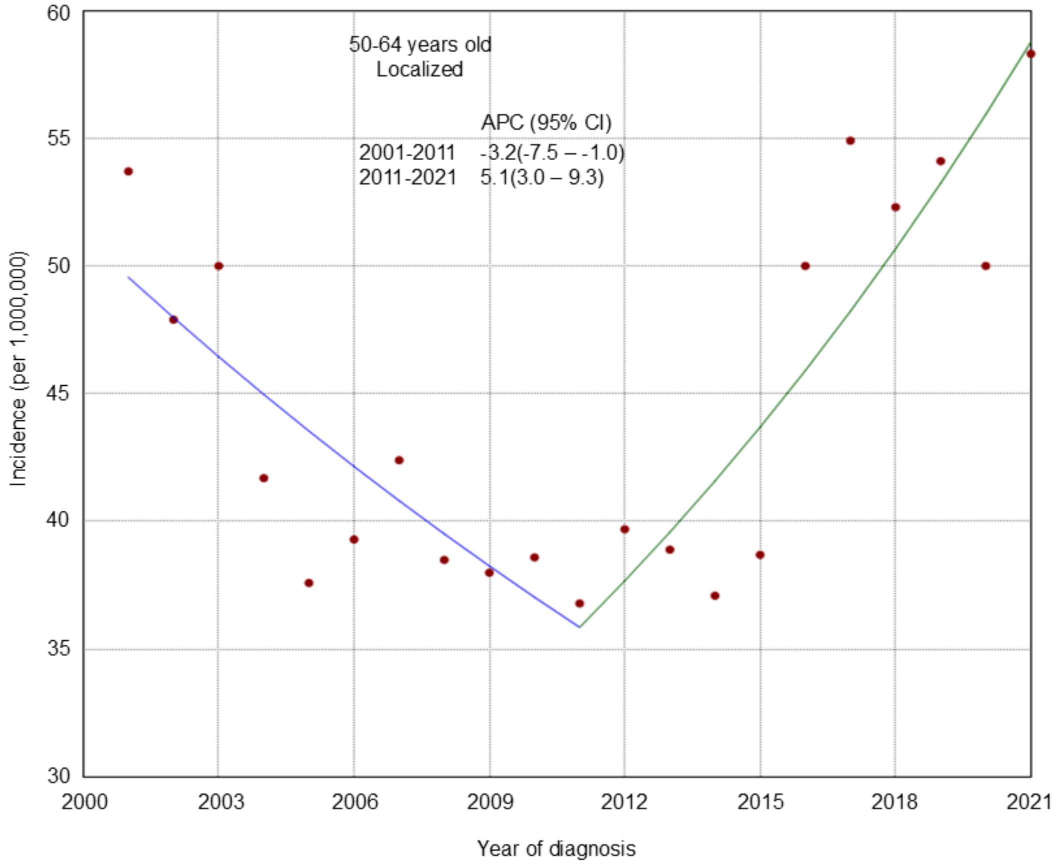

Supplemental Figure 2. B.

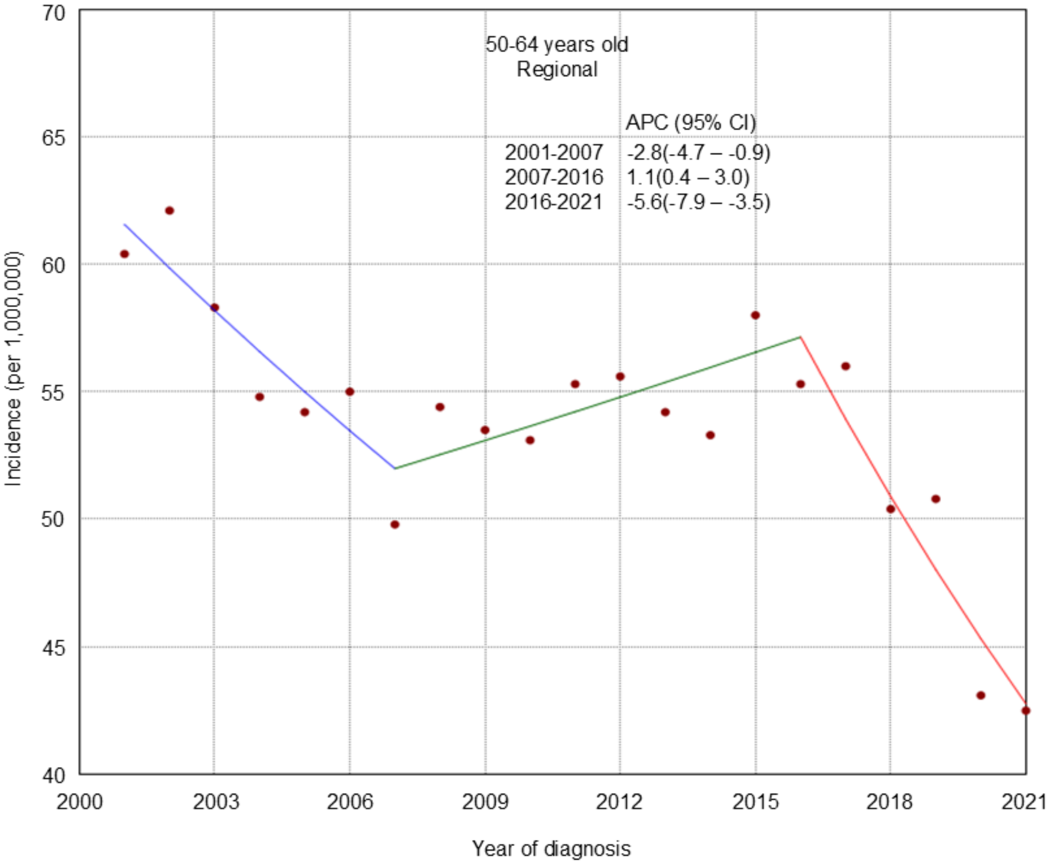

Supplemental Figure 2. C.

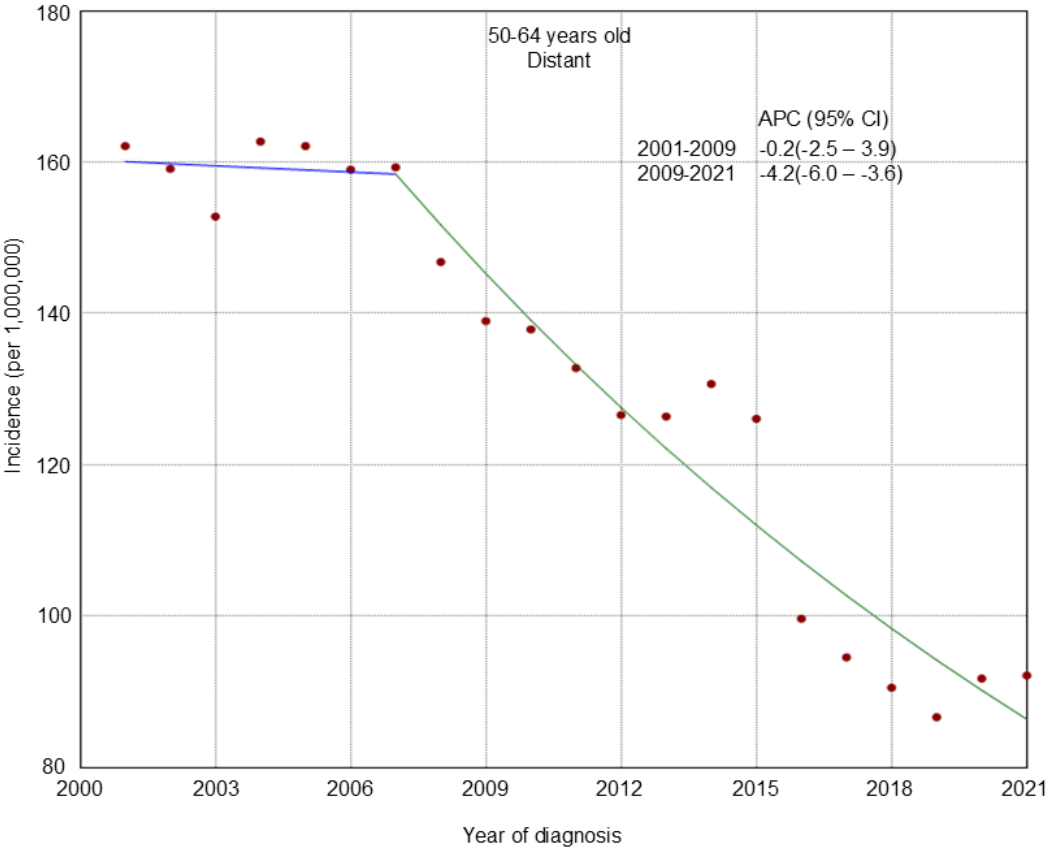

Supplemental Figure 3. A.

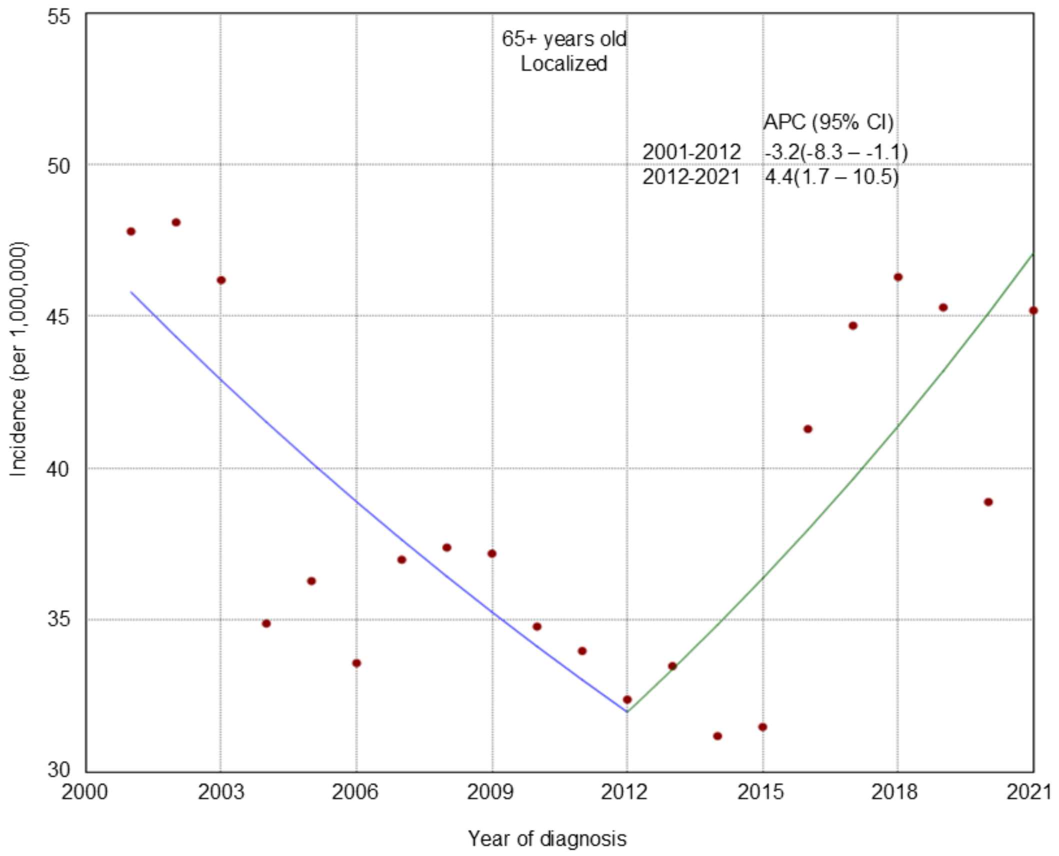

Supplemental Figure 3. B.

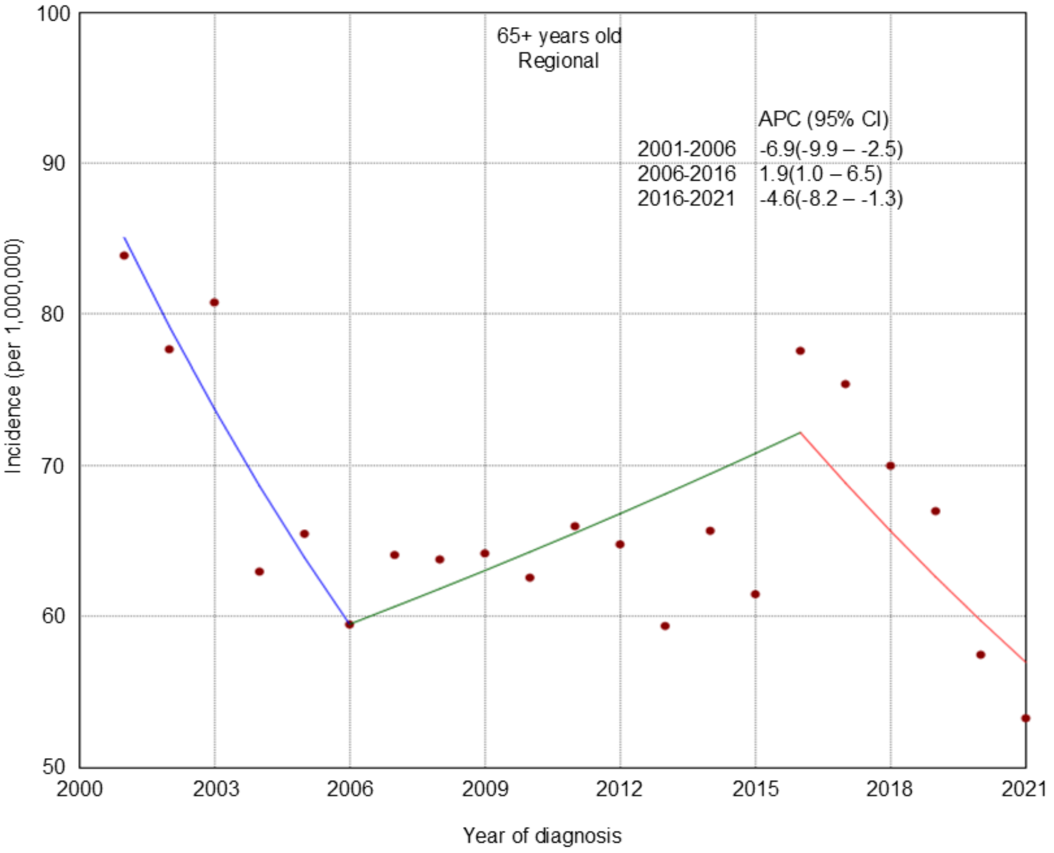

Supplemental Figure 3. C.

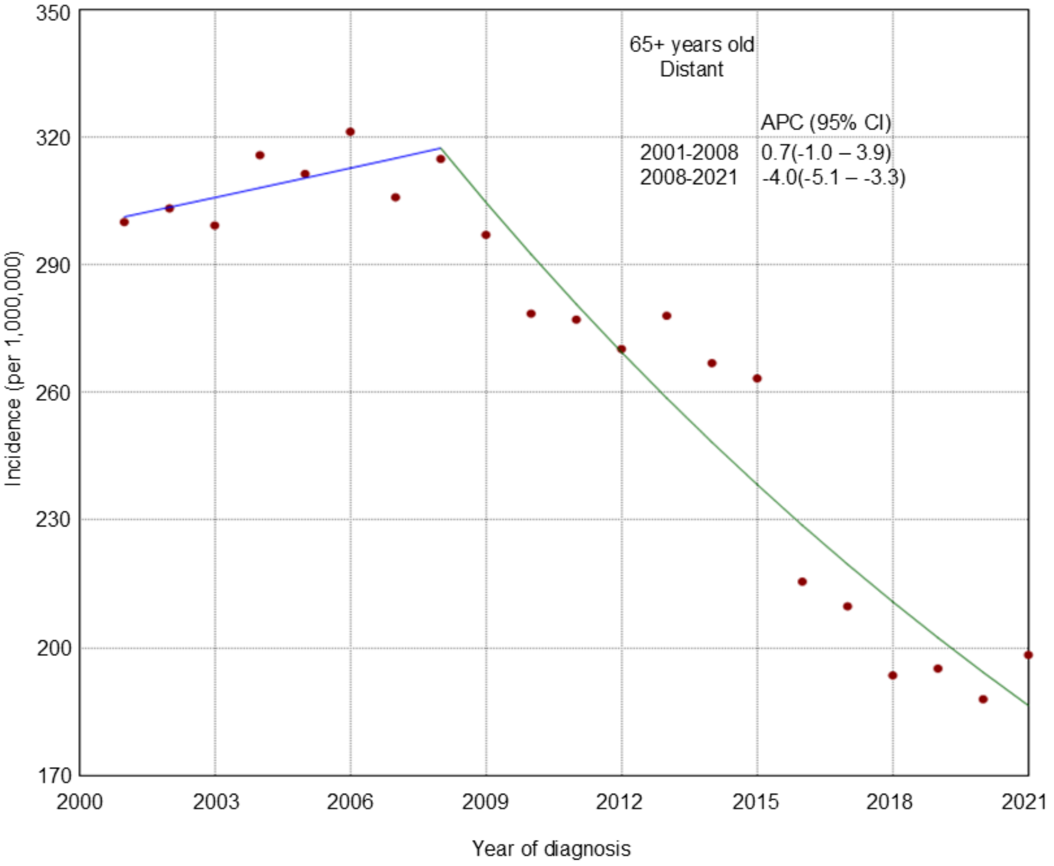

Supplemental Figure 4. A.

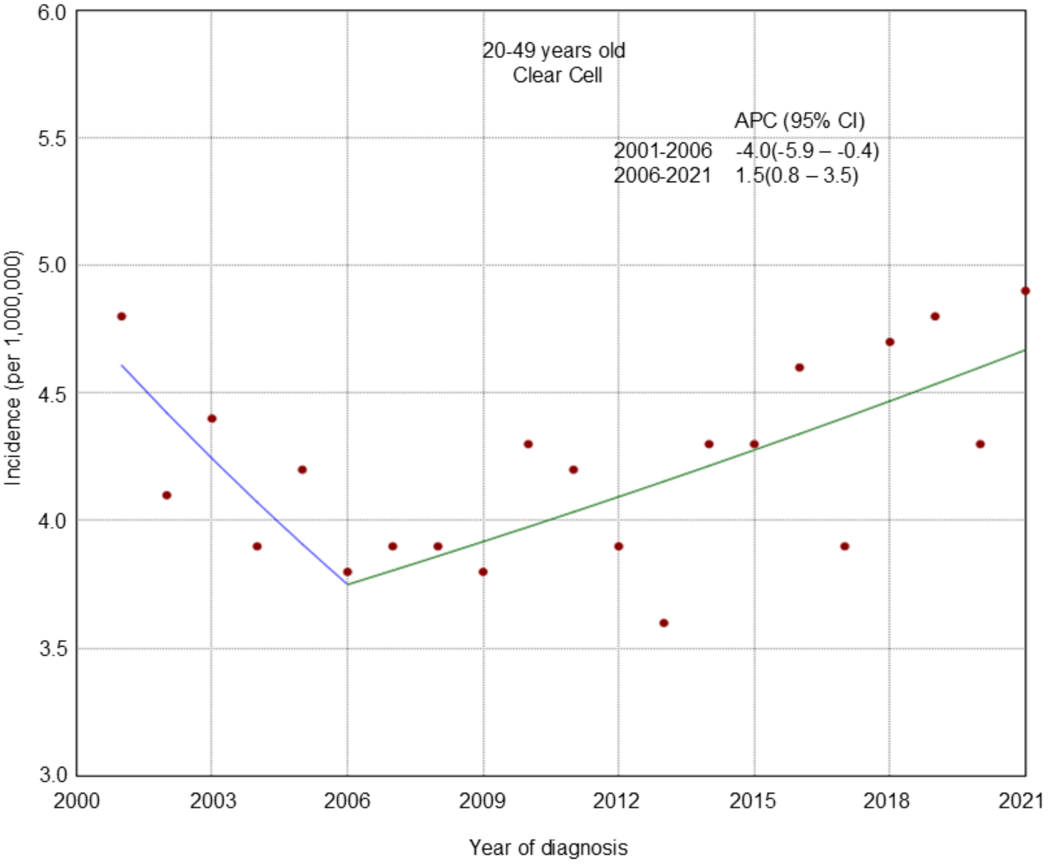

Supplemental Figure 4. B.

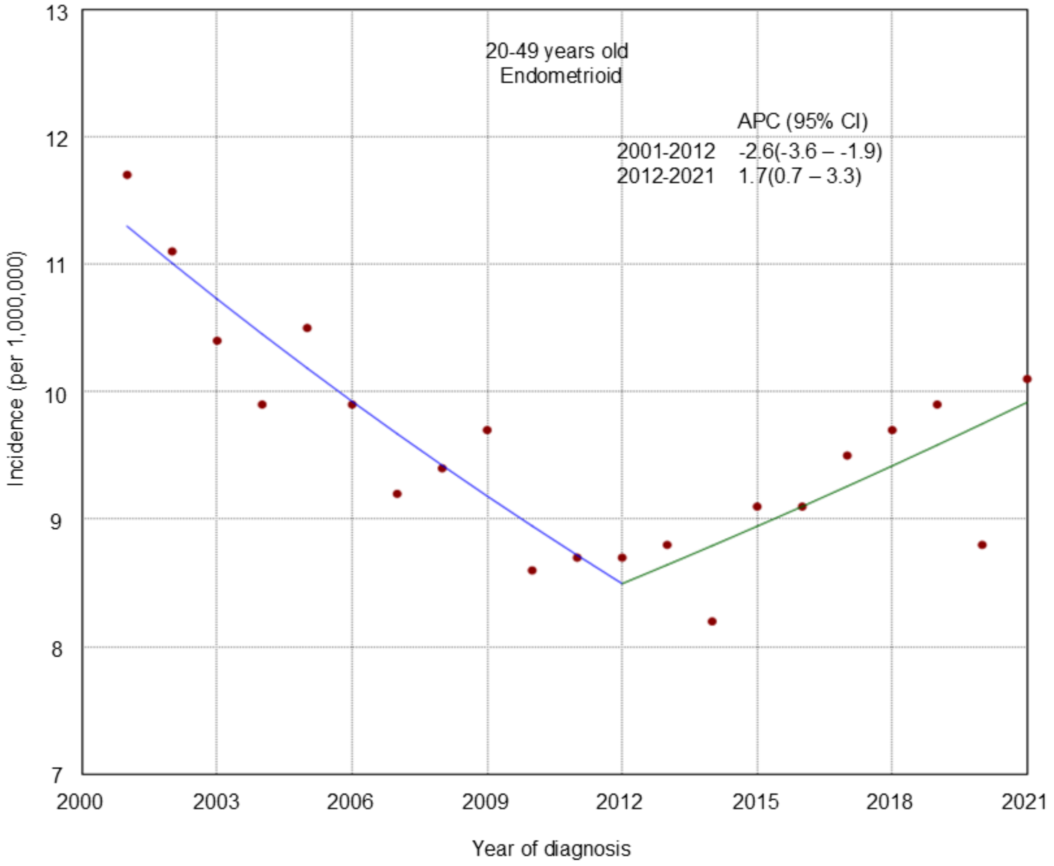

Supplemental Figure 4. C.

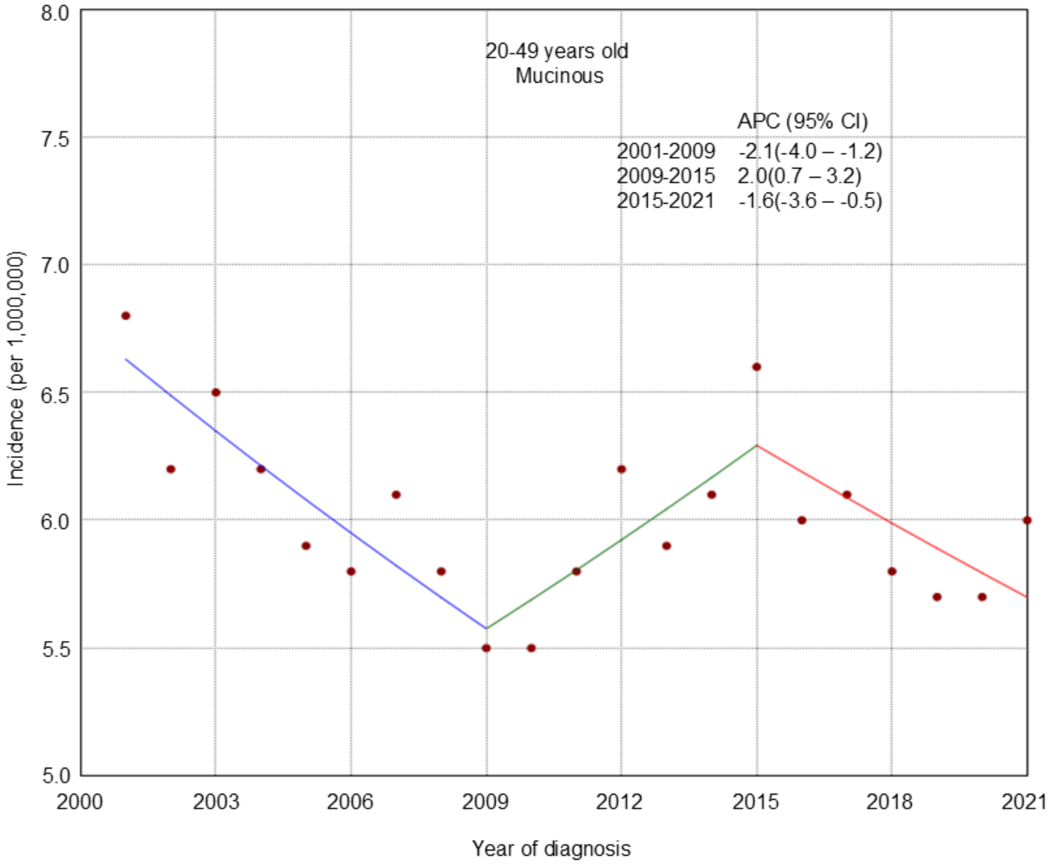

Supplemental Figure 4. D.

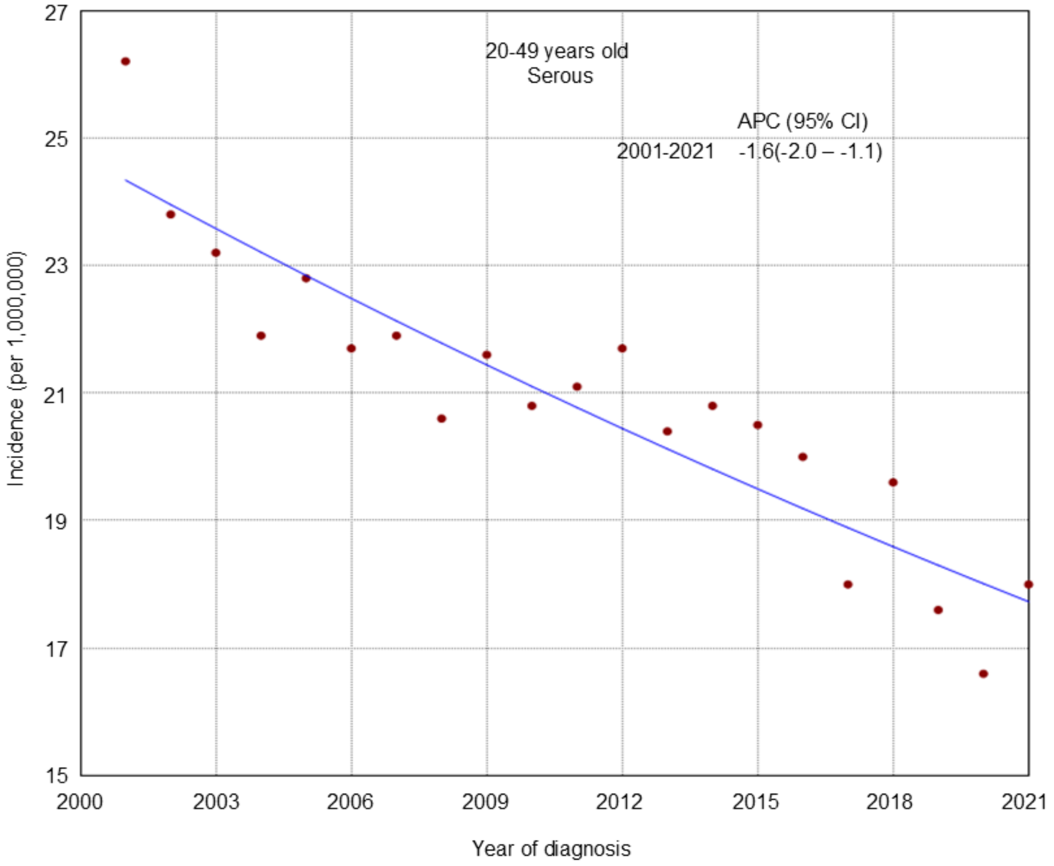

Supplemental Figure 5. A.

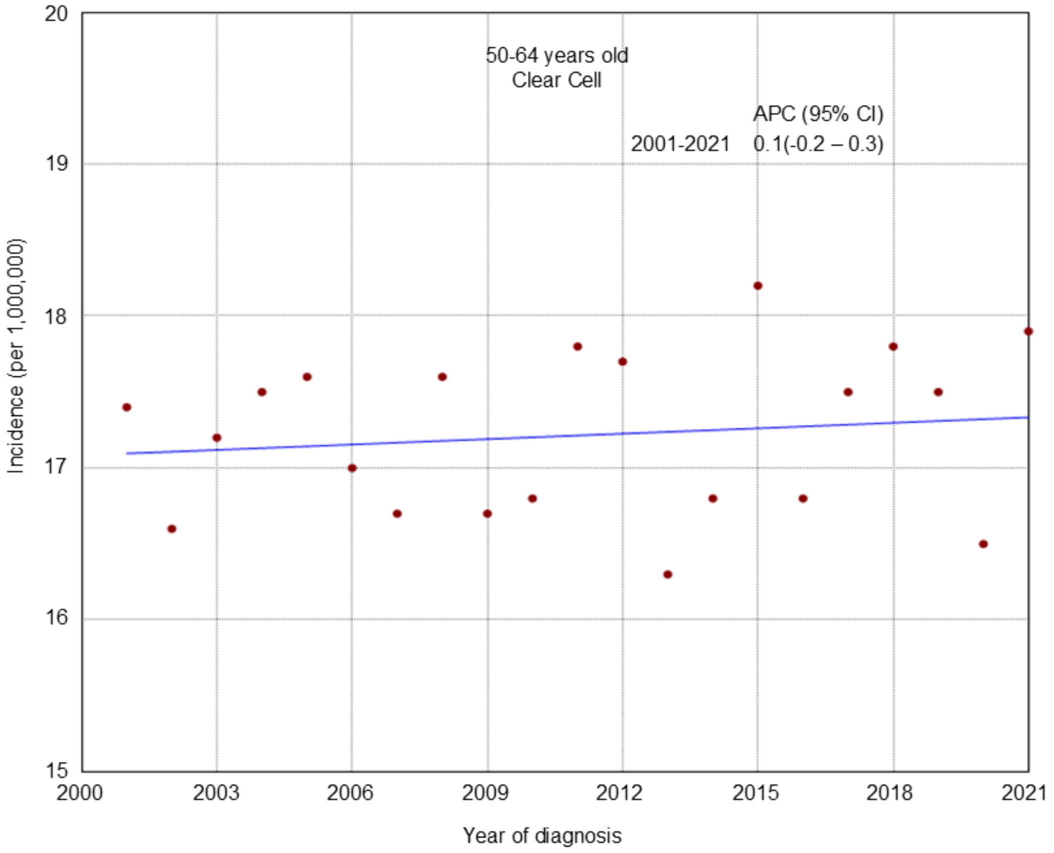

Supplemental Figure 5. B.

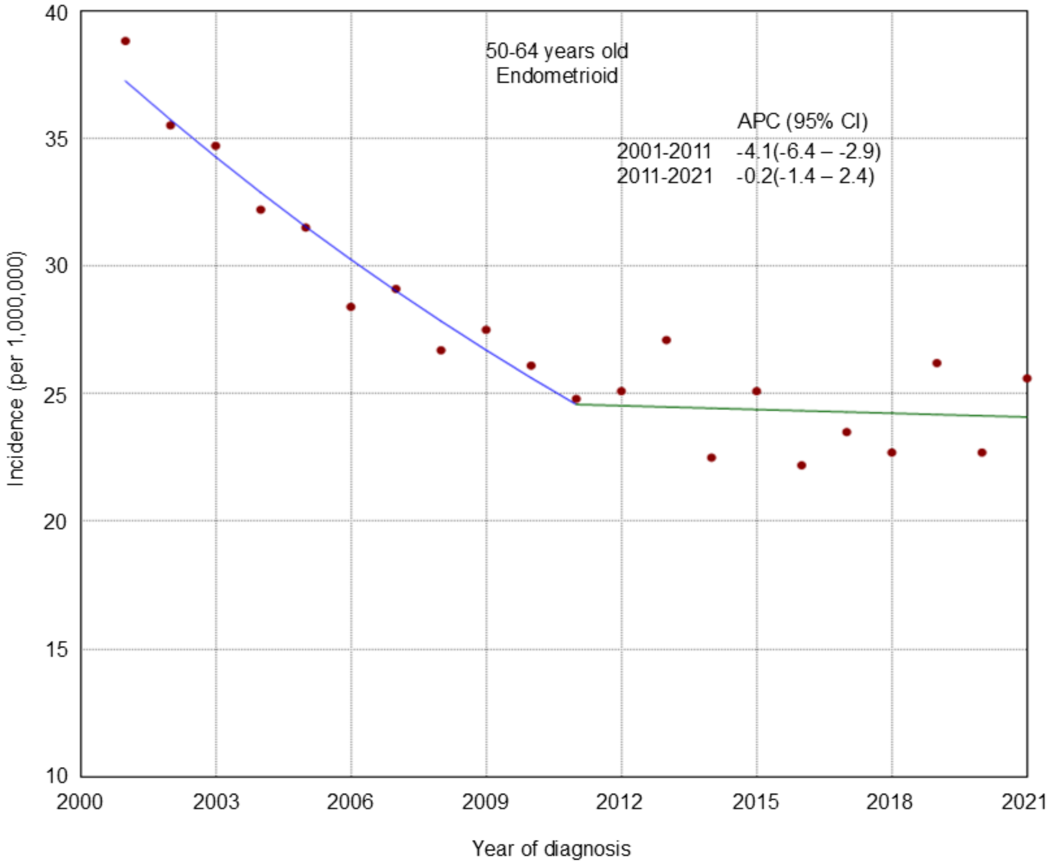

Supplemental Figure 5. C.

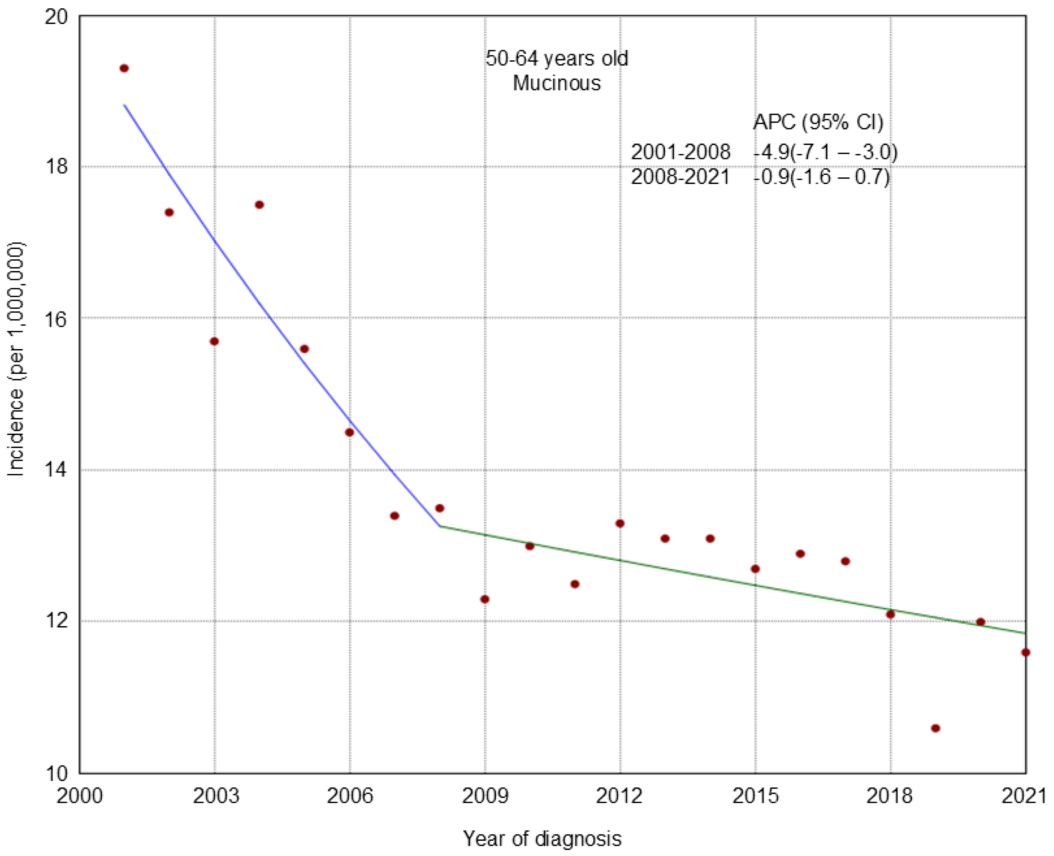

Supplemental Figure 5. D.

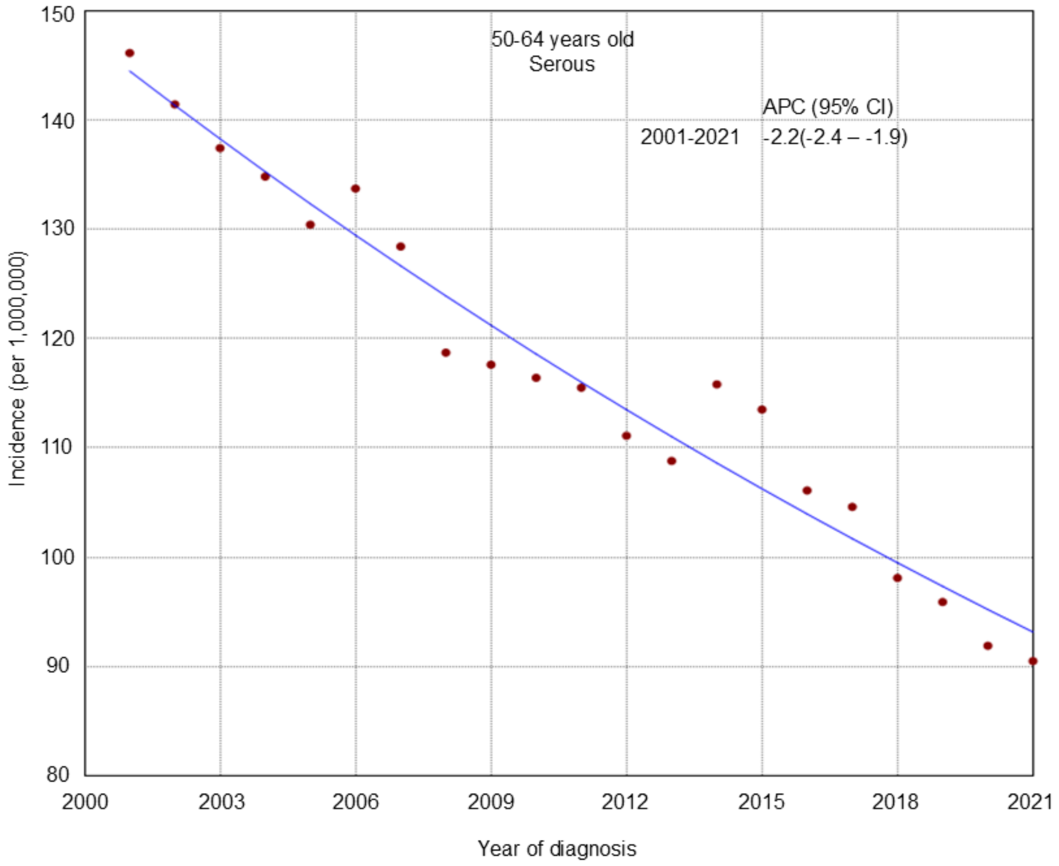

Supplemental Figure 6. A.

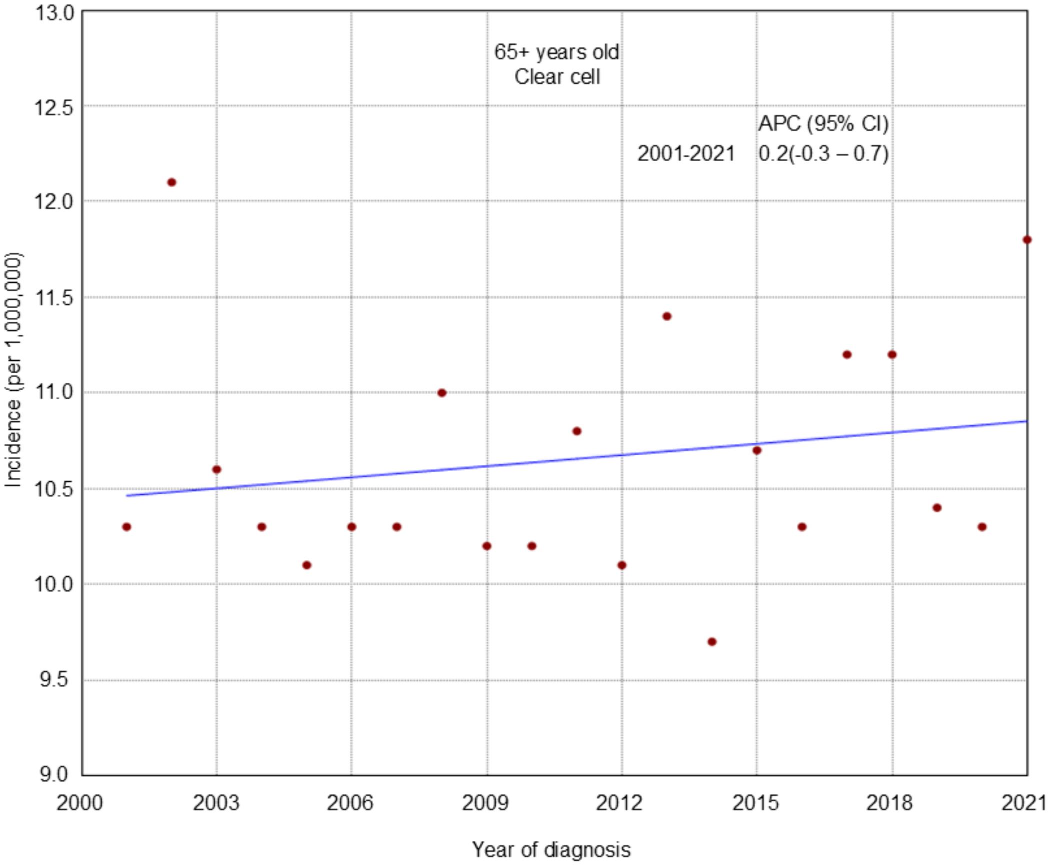

Supplemental Figure 6. B.

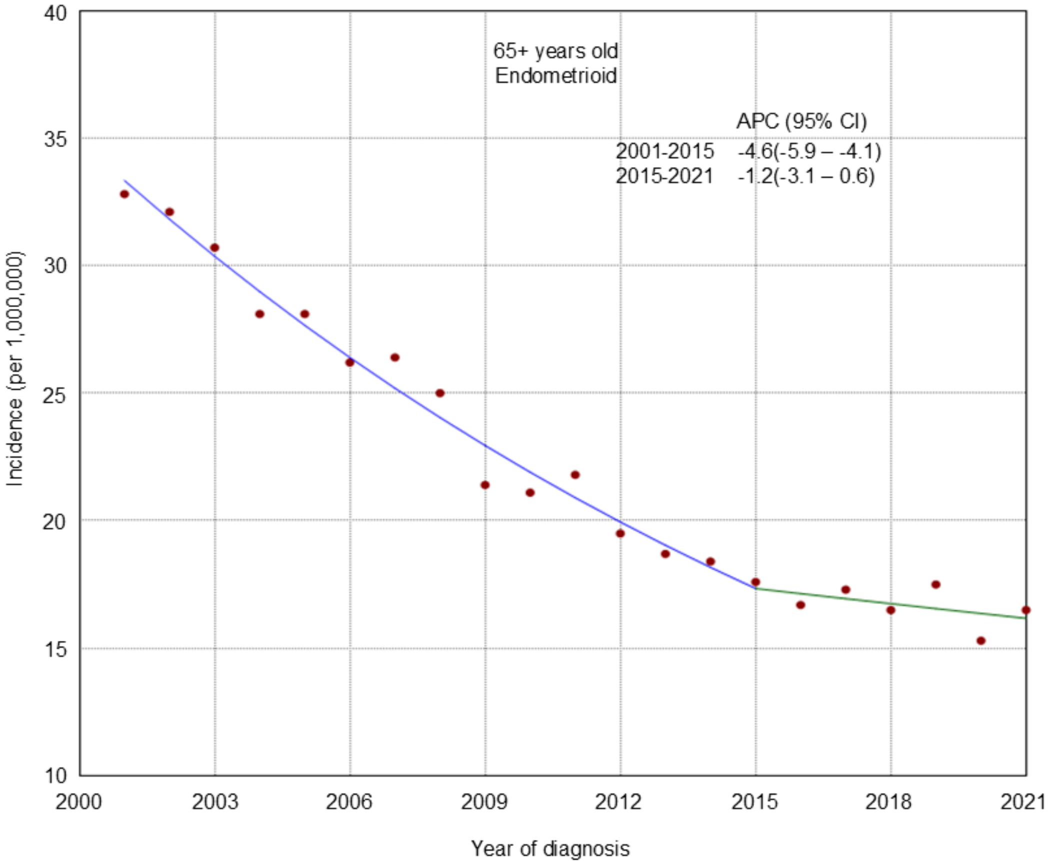

Supplemental Figure 6. C.

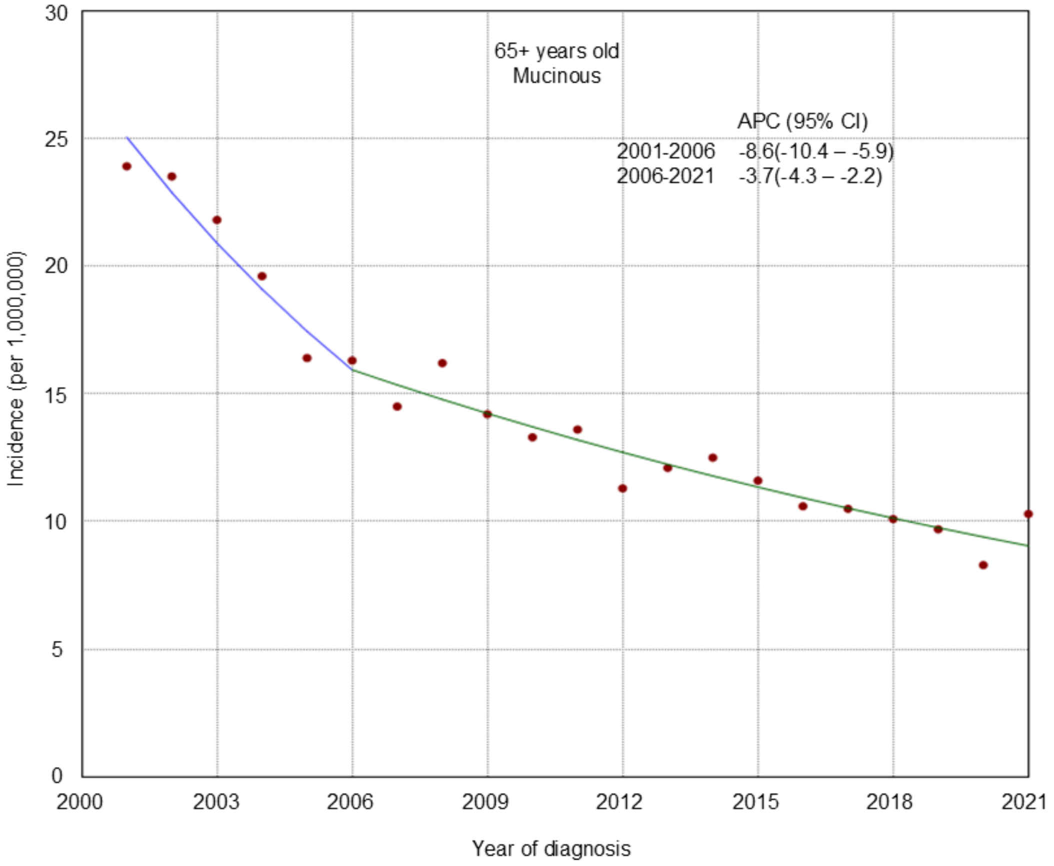

Supplemental Figure 6. D.

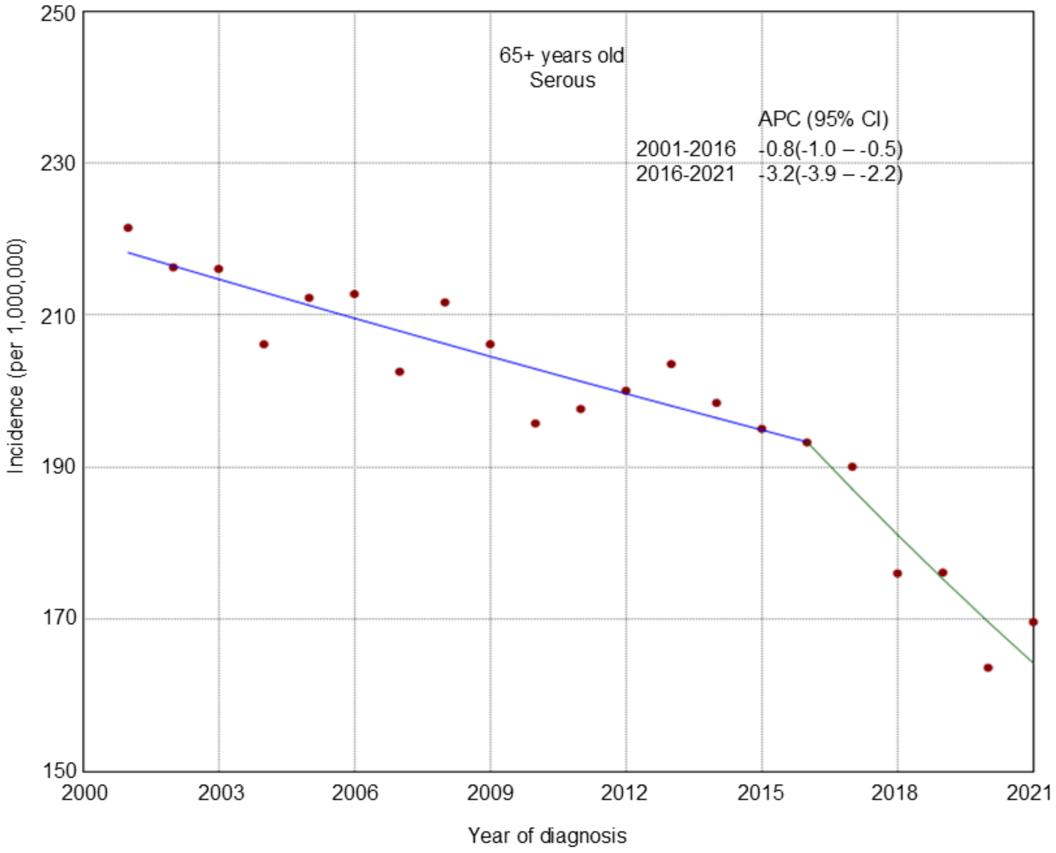

Supplement: Supplementary file 1 [file cancers-17-02119-s001.zip › cancers-3696330-supplementary.pdf]
